# Clinical markers of post-Chikungunya chronic inflammatory joint disease: A Brazilian cohort

**DOI:** 10.1371/journal.pntd.0011037

**Published:** 2023-01-06

**Authors:** Carolina dos Santos Lázari, Mariana Severo Ramundo, Felipe ten-Caten, Clarisse S. Bressan, Ana Maria Bispo de Filippis, Erika Regina Manuli, Isabella de Moraes, Geovana Maria Pereira, Marina Farrel Côrtes, Darlan da Silva Candido, Alexandra L. Gerber, Ana Paula Guimarães, Nuno Rodrigues Faria, Helder I. Nakaya, Ana Tereza R. Vasconcelos, Patrícia Brasil, Gláucia Paranhos-Baccalà, Ester Cerdeira Sabino

**Affiliations:** 1 Hospital das Clínicas, Faculdade de Medicina da Universidade de São Paulo, São Paulo, Brazil; 2 Fleury Medicina e Saúde, São Paulo, Brazil; 3 Departamento de Moléstias Infecciosas e Parasitárias, Faculdade de Medicina da Universidade de São Paulo, São Paulo, Brazil; 4 Instituto de Medicina Tropical, Faculdade de Medicina da Universidade de São Paulo, São Paulo, Brazil; 5 Instituto Nacional de Infectologia Evandro Chagas, Fundação Oswaldo Cruz, Fiocruz, Rio de Janeiro, Brazil; 6 Open Innovation and Partnerships, bioMérieux SA, Lyon, France; 7 Department of Zoology, University of Oxford, Oxford, United Kingdom; 8 LABINFO, Laboratório Nacional de Computação Científica, Petrópolis, Rio de Janeiro, Brazil; 9 MRC Centre of Global Infectious Disease Analysis, Jameel Institute for Disease and Emergency Analytics, Imperial College of London, London, United Kingdom; 10 Scientific Platform Pasteur, Universidade de São Paulo, São Paulo, Brazil; 11 Hospital Israelita Albert Einstein, São Paulo, Brazil; 12 Instituto Todos pela Saúde, São Paulo, Brazil; NIAID Integrated Research Facility, UNITED STATES

## Abstract

**Background:**

Chikungunya-fever (CHIKF) remains a public health major issue. It is clinically divided into three phases: acute, post-acute and chronic. Chronic cases correspond to 25–40% individuals and, though most of them are characterized by long-lasting arthralgia alone, many of them exhibit persistent or recurrent inflammatory signs that define post-Chikungunya chronic inflammatory joint disease (pCHIKV-CIJD). We aimed to identify early clinical markers of evolution to pCHIKV-CIJD during acute and post-acute phases.

**Methodology/Principal findings:**

We studied a prospective cohort of CHIKF-confirmed volunteers with longitudinal clinical data collection from symptoms onset up to 90 days, including a 21-day visit (D21). Of 169 patients with CHIKF, 86 (50.9%) completed the follow-up, from whom 39 met clinical criteria for pCHIKV-CIJD (45.3%). The relative risk of chronification was higher in women compared to men (RR = 1.52; 95% CI = 1.15–1.99; FDR = 0.03). None of the symptoms or signs presented at D0 behaved as an early predictor of pCHIKV-CIJD, while being symptomatic at D21 was a risk factor for chronification (RR = 1.31; 95% CI = 1.09–1.55; FDR = 0.03). Significance was also observed for joint pain (RR = 1.35; 95% CI = 1.12–1.61; FDR = 0.02), reported edema (RR = 3.61; 95% CI = 1.44–9.06; FDR = 0.03), reported hand and/or feet small joints edema (RR = 4.22; 95% CI = 1.51–11.78; FDR = 0.02), and peri-articular edema observed during physical examination (RR = 2.89; 95% CI = 1.58–5.28; FDR = 0.002). Furthermore, patients with no findings in physical examination at D21 were at lower risk of chronic evolution (RR = 0.41, 95% CI = 0.24–0.70, FDR = 0.01). Twenty-nine pCHIKV-CIJD patients had abnormal articular ultrasonography (90.6% of the examined). The most common findings were synovitis (65.5%) and joint effusion (58.6%).

**Conclusion:**

This cohort has provided important insights into the prognostic evaluation of CHIKF. Symptomatic sub-acute disease is a relevant predictor of evolution to chronic arthritis with synovitis, drawing attention to joint pain, edema, multiple articular involvement including small hand and feet joints as risk factors for chronification beyond three months, especially in women. Future studies are needed to accomplish the identification of accurate and early biomarkers of poor clinical prognosis, which would allow better understanding of the disease’s evolution and improve patients’ management, modifying CHIKF burden on global public health.

## Author summary

Chikungunya fever (CHIKF) is a vector-borne viral disease first described in 1952 in Africa, which recently reached the Americas, where it then originated epidemics of unprecedented magnitude. Its acute phase is characterized by fever associated with joint pain and edema, which resolve in about seven days for most patients. However, 25–40% of these patients develop chronic musculoskeletal and arthritic symptoms, which may be incapacitating and lead to permanent joint damage. We have conducted a prospective longintudinal cohort of CHIKF confirmed individuals, in Brazil, which aimed to identify clinical early markers of evolution to post-Chikungunya chronic inflammatory joint disease (pCHIKV-CIJD) after 90 days, using objective physical examination to define pCHIKV-CIJD. We have also performed joint ultrasonography to improve evaluation of chronic arthritis. We found that 45.3% of patients who completed the follow-up met criteria for pCHIKV-CIJD. Women were at higher risk of chronification, as well as individuals who remain symptomatic 21 days after the onset of symptoms. Abnormal ultrasonography results were seen in 90.6% of examined pCHIKV-CIJD patients, in whom synovitis and joint effusion were the most commom songraphic signs, affecting mostly ankles and knees. The adoption of objective criteria to define pCHIKV-CIJD is crucial to estimate accurately the proportion of patients who evolve to chronic rheumatism, and to indentify early risk factors to this outcome, which may add important information to tailor therapeutic strategies for this particular population. It may also help to understand the burden of CHIKF in developing countries, measuring either its impact in individual’s quality of life, or its communitary repercussion after widespread outbreaks.

## Introduction

Chikungunya fever (CHIKF) was first described in 1952 and was then responsible for outbreaks in Africa and Southeast Asia [1]. Chikungunya virus (CHIKV) circulation was first detected in the Americas in 2013. Since then, it originated epidemics of unprecedented magnitude, with over 2.6 million reported cases up to 2017 [2–5].

Since 2014, when local transmission was confirmed in Brazil [6], the virus has caused epidemics in several states [7]. Until 2019, cumulative incidence ranged from less than 10 cases/100,000 inhabitants in southern states, to 1707 cases/100,000 inhabitants in the Northeast of the country. Rio de Janeiro stands out as one of the states with the highest incidence, 869/100,000 inhabitants [8].

CHIKF is characterized by fever associated with joint pain and edema usually beginning three days after an infected mosquito bite [2,9,10] and leading to symptoms resolution in about seven days for most patients. However, 25–40% of these patients experience chronic implications. CHIKF is clinically divided into three phases: acute (from symptoms onset up to 21 days), post-acute (from 21 days to the end of the third month) and chronic (after three months). Although most chronic cases are characterized by long-lasting arthralgia alone, many of them exhibit persistent or recurrent inflammatory signs that define arthritis, enthesitis or tenosynovitis, with variable levels of inflammatory activity and severity of symptoms [2,9–11].

Long-term manifestations after CHIKV infection may present as musculoskeletal disorders (MSD), when there is no direct joint involvement, or chronic inflammatory rheumatisms (CIR), in which multiple joints are affected. MSD are far more frequent (95% of chronic cases) than CIR, but the latter have poorer functional prognosis, as they always imply polyarthritis, which may be incapacitating. Patients with CIR may meet previously estabilished criteria for rheumatoid (RA) arthritis or spondyloarthritis (SA); when they do not, the presentation is described as undifferentiated polyarthritis, which is often depicted as post-Chikungunya chronic inflammatory joint disease (pCHIKV-CIJD) [11,12]. The adoption of objective and homogeneous criteria to define chronic disease after CHIKV infection is crucial to understand better its natural history, improving the accuracy not only to estimate the proportion of patients who evolve chronically, but also to indentify early risk factors to this outcome, which may add important information to tailor therapeutic strategies.

Chronic polyarthritis associated with CHIKF may be vastly incapacitating [13]. In spite of pCHIKV-CIJD major impact on the individual’s quality of life (QOL), potentially overloading public health systems and implying direct and indirect economic losses, clinical-epidemiological studies covering the burden of chronic disease on the Brazilian population are still incipient. Therefore, the present study established a prospective cohort of symptomatic volunteers with confirmed CHIKF, followed-up for 90 days, to explore potential clinical early predictors of chronic disease.

## Methods

### Ethics statement

This study has been approved by the ethical review board of the Faculdade de Medicina da Universidade de São Paulo, under the number CAAE 71611417.9.1001.0065 and appreciation number 2.262.437. All volunteers were adults (18 years or older), leggaly capable and have signed a written informed consent form.

### Study design and procedures

The study consisted of a prospective longitudinal cohort of febrile adults enrolled in a specialized outpatient facility, part of the National Infectious Diseases Institute of Fiocruz, in Rio de Janeiro city, Brazil.

The sample size of 180 volunteers with confirmed CHIKF was calculated for estimating the prevalence rate of pCHIKV-CIJD with maximum marginal error of one quarter of the proportion, stating the expected prevalence of chronic disease in 25%, as described by Hajian-Tilaki (2011) [14].

All patients aged 18 years or older who attended the facility from April to August 2019 were assessed for eligibility with the following inclusion criteria: fever >38.5°C up to 7 days long and arthralgia not explained by other medical conditions. After informed consent, all of them were examined by a physician. Blood samples were drawn to perform CHIKV RT-PCR, serology, and next-generation sequencing (NGS). Patients with either detected RT-PCR or reactive IgM were considered confirmed cases of CHIKF.

Follow-up consisted in two visits, around days 21 (D21) and 90 (D90) after inclusion, in which blood samples were collected and patients were assessed for inflammatory joint pain, number of affected joints, articular edema, heat and/or erythema and/or morning stiffness over 30 minutes. On D90, considering physical examination, patients where assigned to groups “pCHIKV-CIJD” or “non- pCHIKV-CIJD”, based on criteria postulated by French and Brazilian guidelines (Table 1) [11,12]. Volunteers classified to the former group also underwent ultrasonography (US) examination of the most affected joint, in order to confirm and characterize arthritis.

**Table 1:** Criteria for classification of post–Chikungunya chronic inflammatory joint disease (pCHIKV–CIJD).

More than 4 joints with arthritis (A) **AND**
Symptom duration equal to or greater than 6 weeks **AND**
No alternative diagnosis (B)
**A. Arthritis = at least 1 inflammation criteria**
Synovitis*AND/OR
Heat and/or erythema on the joint **AND/OR**
Morning stiffness for longer than 30 minutes **AND/OR**
Inflammatory pain (improves with exercise and worsens with rest or during the night)
**B. Patient does not meet the criteria for rheumatoid arthritis or spondyloarthritis**

*Must be distinguished from joint swelling without synovitis.

Socio-demographic, clinical and pathological data were managed by a software developed specifically for the study.

Pregnant women were excluded, as well as volunteers with pre-existing conditions leading to immunodeficiency or who had used any systemic immunosuppressive drugs in the last 30 days.

### Laboratory specific tests

CHIKV RNA detection was performed by RT-PCR using a commercially available kit, ZDC molecular assay (Bio-Manguinhos, Brazil) and seric anti-CHIKV IgM antibodies were measured using commercial ELISA (Euroimmun, Germany), both according to the manufacturer’s instructions [15,16].

### CHIKV genome sequencing and phylogenetic analysis

Total RNA was obtained from whole blood of thirty-three pCHIKV-CIJD and twenty-nine sex and age paired non-pCHIKV-CIJD patients, using MagMAX for Stabilized Blood Tubes RNA Isolation Kit for Tempus tube (Thermo Fisher Scientific, USA).

The libraries were generated through TruSeq Stranded Total RNA Library Prep with RiboZero Gold (Illumina, USA), loaded onto NextSeq 500/550 High Output Kit v2.5 (150 Cycles), and sequenced using NextSeq 550 System (Illumina).

Reads were taxonomically classified with the Kraken 2 tool (v2.0.9) [17], to identify sequences referring to CHIKV present in blood samples and the results were visualized in the Pavian tool [18]. The Metacompass (v1.12) software was used to align reads to the CHIKV reference genome (KP164568) and generate the consensus sequences [19].

All the FASTA sequences generated in the present study were already available at GenBank (https://www.ncbi.nlm.nih.gov/genbank/) (see S1 Table).

To compose the dataset for phylogenetic analysis, all CHIKV sequences from Brazil with genome coverage above 5 Kb were downloaded from the Virus Pathogen Research database (ViPR, www.viprbrc.org). The Chikungunya Typing Tool (https://www.genomedetective.com/app/typingtool/chikungunya/) was used to identify the CHIKV genotype. Alignment, visual inspection and editing of sequences were performed using AliView 1.23 [20]. Quality control was performed using TempEst v1.5.3 [21] and sequences with a residual over 0.003 were removed from the alignment. Our final dataset was composed of 165 CHIKV genome sequences downloaded from ViPR and the 22 novel CHIKV genome sequences from this study. A maximum likelihood phylogeny was inferred using IQTREE 2 [22] under the best model, GTR + F + R4, selected by ModelFinder [23] implemented in the IQTree-2 platform. No recombination signal was identified using RDP4 [24].

### Statistical analysis

Univariate analysis was performed using Fisher’s Exact Test and Chi-squared Test for categorical variables and Wilcoxon-Mann-Whitney for continuous variables. The results are reported as Risk Ratio and adjusted p-value (False Discovery Ratio—FDR). The statistical analysis was performed in the R software version 4.1.1. FDR below 0.05 was considered statistically significant.

## Results

### Cohort overview

**Fig 1:**
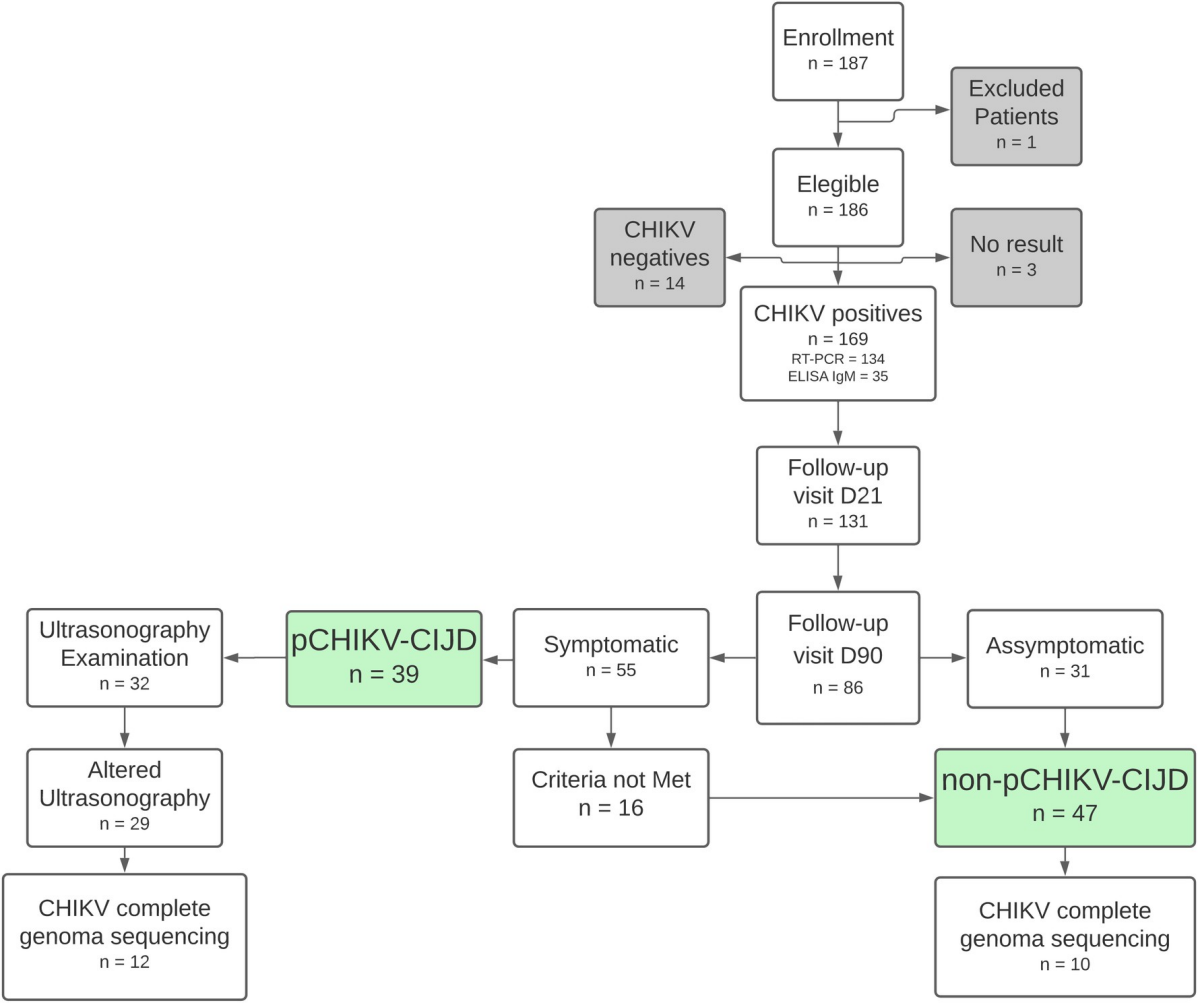
Flowchart showing the progress of the CHIKF cohort. Gray frames represent patients who were excluded from the analysis. Green frames represent the two study groups analyzed. n = number of patients; D21 = Follow–up visit 21 days after inclusion; D90 = Follow–up visit 90 days after inclusion; pCHIKV–CIJD = post–Chikungunya chronic inflammatory joint disease; non–pCHIKV–CIJD = patients who did not met criteria of pCHIKV–CIJD.

Based on the inclusion criteria established for the study, 187 volunteers with fever and joint pain were recruited. One volunteer was excluded (did not sign the informed consent) and 169 had CHIKF diagnosis confirmed, 134 by RT-qPCR and 35 by IgM detection by ELISA. The median time from symptoms onset to inclusion of confirmed cases was three days (see S2 Table). Of these169 CHIKF-confirmed, 131 patients returned for D21 visit. Of the 86 patients who returned for D90 visit, 55 remained symptomatic of which 39 (45.3%; 95% CI = 34.7%-56.4%) met clinical criteria (Table 1) and were classified as pCHIKV-CIJD. Of the pCHIKV-CIJD volunteers, 32 underwent ultrasonography examination and 29 (90.6%) of them showed some alteration. The 16 symptomatic patients on D90 who did not meet the criteria for classification of pCHIKV-CIJD, along with the 31 patients who were asymptomatic were classified as non-pCHIKV-CIJD, establishing the two comparison groups of this study (Fig 1). Data from each of the 169 CHIKV-positive patients can be found in S1 Table (see S1 Table).

### Socio-demographic and clinico-pathological data

Among the 169 CHIKF-confirmed patients, 68.0% (115/169) were female, the median age was 41 years old (19–83 years old), 41.4% (70/169) had complete high school or incomplete undergraduate course, 55.0% (93/169) declare themselves white and 39.1% (66/169) had a family income of up to R$1,874.00. Comparing pCHIKV-CIJD to non-pCHIKV-CIJD groups, no difference was found regarding age (FDR = 0.84), level of education (FDR = 0.06), race (FDR = 0.42) or family income (FDR = 0.28). However, sex seemed to be significantly associated with pCHIKV-CIJD, as 34 of 39 patients in this group (87.2%) were women compared with 27 of 47 (57.5%) in the non-pCHIKV-CIJD group (FDR = 0.03). Moreover, the univariate analysis showed that the relative risk of chronification was higher in women compared to men (RR =: 1.52; 95% CI = 1.15–1.99; FDR = 0.03) (Table 2).

**Table 2:** Socio–demographic data of volunteers with confirmed CHIKF.

	CHIKF-confirmed volunteers n or median (% or variation)	pCHIKV-CIJD volunteers n or median (% or variation)	non-pCHIKV-CIJD volunteers n or median (% or variation)	RR	95% CI	FDR

Total	169 (100.0)	39 (100.0)	47 (100.0)			
**Age Median**	41 (19–83)	46 (22–80)	50 (21–79)	-	-	0.84
**Sex**						
Female	115 (68.0)	34 (87.2)	27 (57.4)	**1.52**	**1.15–1.99**	**0.03**
Male	54 (32.0)	5 (12.8)	20 (42.6)			
**Level of education**						
No formal education	1 (0.6)	0 (0.0)	1 (2.1)	-	-	0.06
Elementary school, initial years (up to 4th year)	19 (11.2)	6 (15.4)	4 (8.5)			
Elementary school (up to 8th or 9th grade) or incomplete high school	26 (15.4)	6 (15.4)	10 (21.3)			
Complete high school or incomplete undergraduate course	70 (41.4)	12 (30.8)	20 (42.6)			
Higher education, complete undergraduate course	43 (25.4)	9 (23.1)	12 (25.5)			
Postgraduate Lato-sensu or Stricto-sensu	9 (5.3)	8 (20.5)	1 (2.1)			
Unknown	1 (0.6)	0 (0.0)	0 (0.0)			
**Race**						
White	93 (55.0)	20 (51.3)	26 (55.3)	-	-	0.42
Brown	41 (24.3)	15 (38.5)	12 (25.5)			
Black	34 (20.1)	4 (10.2)	9 (19.1)			
Other	1 (0.6)	0 (0.0)	1 (2.1)			
**Family income**						
Up to R$*1,874.00	66 (39.1)	14 (35.9)	20 (42.6)	-	-	0.28
From R$1,874.01 to R$3,748.00	60 (35.5)	14 (35.9)	18 (38.3)			
From R$3,748.01 to R$9,370.00	33 (19.5)	6 (15.4)	7 (14.9)			
From R$9,370.01 to R$18,740.00	8 (4.7)	5 (12.8)	1 (2.1)			
Unknown	2 (1.2)	0 (0.0)	0 (0.0)			

CHIKF: Chikungunya fever; pCHIKV–CIJD: post–Chikungunya chronic inflammatory joint disease; RR: Relative risk; 95 CI: 95% Confidence interval; FDR: False discovery rate

*Brazilian reais. Variables with statistic significance (FDR<0,05) are highlighted in bold.

All 169 patients reported fever and joint pain at D0, as expected once these were inclusion criteria. In turn, headache (117/169; 69.2%), myalgia (110/169; 65.1%), and retro-orbital pain (69/169; 40.8%) were the most common additional symptoms (Table 3) and were combined in 21 (12.4%) patients (see S1A Fig). The most affected joints were knees (130/169; 76.9%), ankles (125/169; 73.9%), and hands (114/169; 67.5%) (see S1B Fig).

**Table 3:** Clinical categorical variables and univariate analysis from CHIKF–confirmed patients at D0 and D21.

	Enrolled n (%)	Follow-up until D21 n (%)	Follow-up until D90 n (%)	pCHIKV-CIJD n (%)	non-pCHIKV-CIJD n (%)	RR^§^	95% CI	FDR^§^
**Total**	169	131	86	39	47	-	-	-
**Early signs and symptoms (from symptoms onset to inclusion)**								
Fever (inclusion criterium)	169 (100.0)	131 (100.0)	86 (100.0)	39 (100.0)	47 (100.0)	1.00	1.00	1.00
Joint Pain (inclusion criterium)	169 (100.0)	131 (100.0)	86 (100.0)	39 (100.0)	47 (100.0)	1.00	1.00	1.00
Peri-articular edema and/or erythema	7 (4.1)	5 (3.8)	4 (4.6)	2 (5.1)	2 (4.2)	1.20	0.17–8.16	1.00
Joint edema and/or erythema	40 (23.7)	33 (25.2)	27 (31.4)	15 (38.5)	12 (25.5)	1.50	0.80–2.82	0.88
**Reported signs and symptoms at inclusion (D0)**								
Joint pain	169 (100.0)	125 (95.4)	86 (100.0)	39 (100.0)	47 (100.0)	1.00	1.00	1.00
Headache	117 (69.2)	93 (71.0)	65 (75.6)	29 (74.4)	36 (76.6)	0.97	0.76–1.23	1.00
Myalgia (back, thighs, calves, arms)	110 (65.1)	81 (61.8)	51 (59.3)	26 (66.7)	25 (53.2)	1.25	0.88–1.77	0.90
Retro-orbital pain	69 (40.8)	50 (38.2)	35 (40.7)	16 (41.0)	19 (40.4)	1.01	0.61–1.69	1.00
Skin rash	54 (31.9)	38 (29.0)	28 (32.5)	15 (38.5)	13 (27.6)	1.39	0.76–2.56	0.96
Conjunctivitis	27 (16.0)	19 (14.5)	13 (15.1)	7 (17.9)	6 (12.8)	1.41	0.51–3.83	1.00
Any edema	25 (14.8)	16 (12.2)	13 (15.1)	7 (17.9)	6 (12.8)	1.41	0.51–3.83	1.00
Arthritis signs (edema, erythema and heat)	10 (5.9)	5 (3.8)	4 (4.6)	3 (7.7)	1 (2.1)	3.62	0.39–33.38	0.93
**Physical examination at inclusion (D0)**								
Any edema	69 (40.8)	55 (42.0)	41 (47.7)	18 (46.2)	23 (48.9)	0.94	0.60–1.47	1.00
Peri-articular edema	42 (24.8)	33 (25.2)	24 (27.9)	11 (28.2)	13 (27.6)	1.02	0.51–2.01	1.00
Articular edema	30 (17.7)	23 (17.6)	19 (22.1)	9 (23.1)	10 (21.3)	1.08	0.49–2.39	1.00
Soft tissue edema (non peri-articular)	1 (0.6)	0 (0.0)	0 (0.0)	0 (0.0)	0 (0.0)	NA	NA—NA	1.00
Any skin rash	101 (59.8)	81 (61.8)	59 (68.6)	27 (69.2)	32 (68.1)	0.99	0.74–1.32	1.00
Diffuse maculo-papular	78 (46.1)	61 (46.6)	46 (53.5)	22 (56.4)	24 (51.1)	1.10	0.74–1.63	1.00
Petechial	5 (3.0)	4 (3.0)	3 (3.5)	1 (2.6)	2 (4.2)	0.60	0.05–6.39	1.00
Hyperchromic	0 (0.0)	0 (0.0)	0 (0.0)	0 (0.0)	0 (0.0)	NA	NA—NA	1.00
Arthritis signs (edema, erythema and heat)	24 (14.2)	14 (10.7)	7 (8.1)	4 (10.3)	3 (6.4)	1.60	0.38–6.75	1.00
**Reported signs and symptoms at D21**								
Any symptom	-	112 (85.5)	73 (84.9)	38 (97.4)	35 (74.5)	**1.31**	**1.09–1.55**	**0.03**
Joint pain	-	109 (83.2)	72 (83.7)	38 (97.4)	34 (72.3)	**1.35**	**1.12–1.61**	**0.02**
Any edema	-	33 (25.2)	20 (23.2)	15 (38.5)	5 (10.6)	**3.61**	**1.44–9.06**	**0.03**
Hands and feet small joints edema	-	29 (22.1)	18 (20.9)	14 (35.9)	4 (8.5)	**4.22**	**1.51–11.78**	**0.02**
Muscular pain	-	30 (22.9)	17 (19.8)	8 (20.5)	9 (19.1)	1.07	0.45–2.51	1.00
Joint stiffness	-	10 (7.6)	7 (8.1)	3 (7.7)	4 (8.5)	0.90	0.21–3.79	1.00
Other joints edema or erythema	-	10 (7.6)	6 (7.0)	4 (10.3)	2 (4.2)	2.41	0.46–12.46	0.99
Skin rash	-	6 (4.6)	2 (2.3)	1 (2.6)	1 (2.1)	1.21	0.07–18.64	1.00
**Physical examination at D21**								
No Findings	-	63 (48.1)	43 (50.0)	11 (28.2)	32 (68.1)	**0.41**	**0.24–0.70**	**0.01**
Peri-articular edema	-	52 (39.7)	34 (39.5)	24 (61.5)	10 (21.3)	**2.89**	**1.58–5.28**	**0.002**
Arthritis signs (edema, erythema and heat)	-	13 (9.9)	11 (12.8)	7 (17.9)	4 (8.5)	2.11	0.66–6.67	0.88
Walking impairment	-	11 (8.4)	9 (10.5)	7 (17.9)	2 (4.2)	4.22	0.92–19.15	0.42
Joint stiffness	-	13 (9.9)	9 (10.5)	6 (15.4)	3 (6.4)	2.41	1.79–4.49	0.92
Skin rash	-	17 (13.0)	7 (8.1)	4 (10.3)	3 (6.4)	1.61	0.38–6.75	1.00
Antalgic position	-	2 (1.5)	2 (2.3)	1 (2.6)	1 (2.1)	1.21	0.64–9.01	1.00
Face edema	-	0 (0.0)	0 (0.0)	0 (0.0)	0 (0.0)	NA	NA—NA	1.00
**Drugs uptaken from symptoms onset to D21** *								
Common analgesics	-	0 (0.0)	0 (0.0)	0 (0.0)	0 (0.0)	NA	NA—NA	1.00
Opioids analgesics	-	43 (32.8)	26 (30.2)	14 (35.9)	12 (25.5)	1,4	0.73–2.67	1.00
Non-steroidal anti-inflammatory drugs	-	0 (0.0)	0 (0.0)	0 (0.0)	0 (0.0)	NA	NA—NA	1.00
Costicosteroids	-	8 (6.1)	6 (7.0)	4 (10.3)	2 (4.2)	2,41	0.46–12.46	1.00
No drugs	-	25 (14.8)	17 (19.8)	3 (7.7)	14 (29.8)	0.26	0.08–0.83	0.09

CHIKF: Chikungunya fever; D0: Inclusion visit; D21: Follow–up visit 21 days after inclusion; D90: Follow–up visit 90 days after inclusion; pCHIKV–CIJD: post–Chikungunya chronic inflammatory joint disease; NA: Not available; RR: Relative risk; 95 CI: 95% Confidence interval; FDR: False discovery rate; § Refers to pCHIKV–CIJD x non–pCHIKV–CIJD groups.

*Other drugs not depicted. Variables with statistic significance (FDR<0,05) are highlighted in bold.

Physical examination at inclusion visit (D0) showed even more signs than reported by patients. Skin rash was present in 101 of 169 (59.8%) patients, predominantly with diffuse maculopapular pattern (78/169; 46.1%), edema was observed in 69 (40.8%) patients, while 24 (14.2%) had visible arthritis signs (edema, erythema and heat) (Table 3).

**Fig 2:**
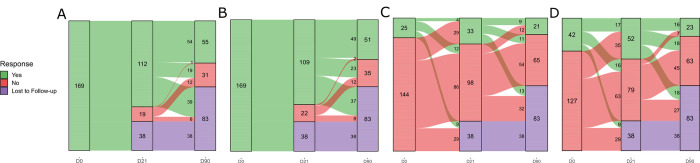
Evoltution of volunteers’ symptoms and signs along the follow–up. **A.** Any symptom. **B.** Joint pain. **C.** Reported joint edema. **D.** Peri–articular edema in physical examination. **Yes:** With symptom; **No:** Without symptom; **Missing:** Patient did not return and was not contacted in the period.

At D21, 112 of the 131 (85.5%) patients who returned to this visit were still symptomatic (Fig 2A). The most common reported symptoms were joint pain (109/131; 83.2%) and edema 33/131; 25.2%). During physical examination, arthritis signs were present in 13 (9.9%) patients, and peri-articular edema stood out for being present in 52 patients (39.7%).

From 55 patients who remained symptomatic at D90, 51 (92.7%) had joint pain, and the main pattern of evolution was persistent pain (49/51; 96.1%), as only two patients reported no pain at D21 and relapsed it at D90 (Fig 2B).

Nonetheless, 29 of 33 patients who reported edema at D21 (87.9%) had not reported this sign at the inclusion visit, as well as 12 who reported the sign on D90 (62.5%) had not reported it on D21 (Fig 2C). The same pattern could be percieved during physical examination: from 52 patients in whom peri-articular edema was recognized at D21, 35 (67.3%) had not presented this sign at D0 (Fig 2D), along with 11 of 13 patients who exhibited arthritis signs.

Concerning risk factors for pCHIKV-CIJD, although any of the symptoms or signs presented at D0 behaved as an early predictor, being symptomatic at D21 was a risk factor for chronification (RR = 1.31; 95% CI = 1.09–1.55; FDR = 0.03) (Table 3). Significance was also individually observed for joint pain (RR = 1.35; 95% CI = 1.12–1.61; FDR = 0.02), reported edema (RR = 3.61; 95% CI = 1.44–9.06; FDR = 0.03) and reported hand and/or feet small joints edema (RR = 4.22; 95% CI = 1.51–11.78; FDR = 0.02). Peri-articular edema observed during physical examination was also significant (RR = 2.89; 95% CI = 1.58–5.28; FDR = 0.002). Furthermore, patients with no findings in physical examination at D21 were at lower risk of chronic evolution (RR = 0.41, 95% CI = 0.24–0.70, FDR = 0.01).

It was also possible to observe that artircular involvement in early stages was more severe in patients who evolved to pCHIKV-CIJD. At D21, pCHIKV-CIJD group had a median of nine affected joints, while non-pCHIKV-CIJD had a median of four (FDR = 0.003) (see S2 Table). Interestingly, although not statiscally significant, patients of the later group tendend to need less symptomatic drugs from onset to D21 than the former (No drug: RR = 0.26; 95% CI = 0.08–0.83; FDR = 0.09) (Table 3). As expected, from D21 to D90, patients who evolved to chronic disease used medication more often than those who resolved the disease earlier, with significance observed for common and opioids analgesics, non-steroidal anti-inflammatory drugs and corticosteroids (see S3 Table).

Neither arthropathy nor arbovirosis (dengue, chikungunya or zika) antecedents behaved as risk factor for chronification (see S3 Table). CHIKV viral load at diagnosis, reflected by RT-PCR cycle threshold (Ct) was not significantly different between groups (see S2 Table).

Thirty-two pCHIKV-CIJD patients underwent ultrasonography examination of the most affected joint and 29 (90.6%) of those had abnormal results. Ankles (14/29; 48.3%) and knees (10/29; 34.5%) were the most frequently abnormal joints, and the most common sonographic findings were synovitis (19/29; 65.5%) and joint effusion (17/29; 58.6%) (see S2 Fig).

### CHIKV genome sequencing and phylogenetic analysis

**Fig 3:**
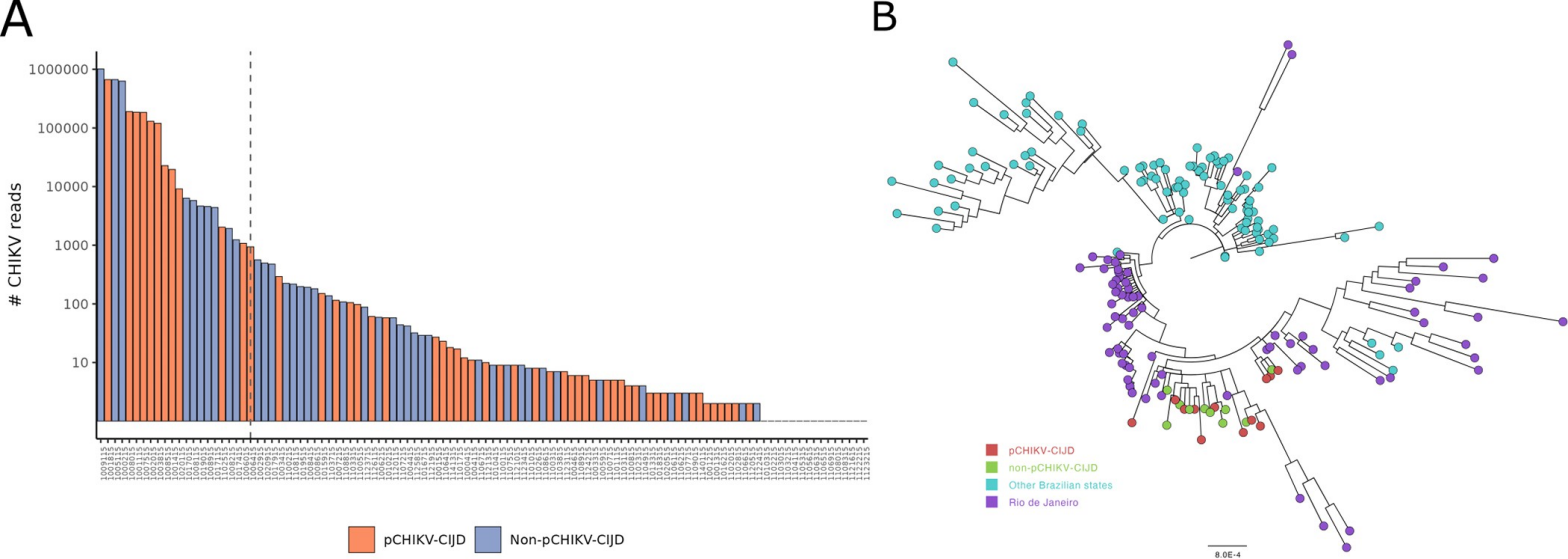
CHIKV genome sequencing and phylogenetic analysis. **A.** Number of reads of CHIKV sequences per sample. To the left of the dotted line are samples with a number of reads enough to cover CHIKV complete genome. **B.** The phylogenetic tree was obtained by comparing pCHIKV–CIJD, non–pCHIKV–CIJD, and other publicly available samples from Rio de Janeiro and other states.

At least one read of the CHIKV genome was detected in 46 samples, including 36 in which at least 100 reads were identified. Complete CHIKV genome was retrieved from 12 pCHIKV-CIJD and 10 non-pCHIKV-CIJD samples (Fig 3A). The size of complete genomic sequences ranged between 11,665 and 11,808 nt.

D0 samples had the highest number of reads of the CHIKV genome, in both groups, due to the higher viral loads reflected by lower cycle thresholds (Cts) (R = -0.84 and p-value = 0.00065). However, it was not possible to identify a clear difference in mean Ct or in the number of CHIKV genome reads between chronic and non-chronic patients.

All 22 sequences belonged to the East-Central-South-African (ECSA) genotype. We could not find any mutations capable of consistently discriminating samples from the two groups and then correlate to chronification (Simmonds’ association index = 0.91, bootstrap = 495/1000) (Fig 3B).

## Discussion

Our study comprises a cohort of laboratory confirmed cases of CHIKF enrolled during a Brazilian regional outbreak, which could criteriously define 39 patients who evolved to pCHIKV-CIJD, representing 45.3% of those who completed the follow-up. Although chronification proportions largely vary in literature [25–30], our findings are very close to those estimated by a recent meta-analysis which managed to minimize heterogeneity, such as different diagnostic criteria for pCHIKV-CIJD and diverse follow-up times [31].

The first published meta-analysis on the issue comprised 5,702 patients from 18 studies and found a pooled prevalence of 40.2% CHIKV-derived chronic inflammatory disease [31]. This was similar to a larger study executed two years later, which pooled more than 6,000 patients from 34 studies, with a non-recovery rate of 43.0%. Nevertheless, any persistent symptoms beyond 3 months were considered as chronic disease and no criteria for pCHIKV-CIJD had been defined [27].

One interesting observation by Rodriguez-Morales and cols [31] was that retrospective studies tend to overestimate this prevalence, as in pooled data from nine prospective studies (2,226 patients), the prevalence of CIJD decreased to 25.3%. Although diagnostic criteria were well established, comparisons to our study are limited because no cohorts from Latin-America were included. In addition to different circulating CHIKV genotypes, diversity in genetic background or immunological response between populations can impose considerable variations in chronification rates.

Another meta-analysis, comprising only cohorts conducted in the Americas, estimated the prevalence of chronic CHIKF at 52.0% in a pooled population of 2,415 individuals, which decreased to 48.0% when only prospective studies were considered. However, these rates also included patients who reported only chronic pain. Only one of the studies was conducted in Brazil [26].

Well-characterized cohorts of confirmed CHIKF are scarce and heterogeneous in Brazil. For instance, in a recent study [32], the authors questioned the patients about the persistence of joint symptoms by telephone contact, after an average 1.5 years since acute illness; 42.5% of them reported joint pain that had last more than 3 months, while 30.7% were still symptomatic at the time of the interview. Nevertheless, one should consider a recall bias in these results, as there has been no clinical evaluation or objective criteria for pCHIKV-CIJD. In 2020, 107 patients from two Brazilian states with confirmed CHIKF were followed for 12-months, 60.7% of whom reported persistent arthralgia, together with at least one of the following: edema, signs of arthritis or tenosynovitis [33,34].

It is worth emphasizing that the proportion of pCHIKV-CIJD estimated in our study regards only to volunteers who completed the follow up, which may introduce a selection bias, as symptomatic patients are more prone to return to medical assistance. Telephone contact with those patients who missed D90 visit was successful for 29 patients, only three of whom still reported joint pain with signs of arthritis. Including these volunteers would decrease the prevalence of CIJD to 36.5% (42/115).

Regardless of this limitation, our study tried to reduce possible biases, not only using previously established clinical criteria, but also confirming arthritis of most patients with ultrasonography. If only patients with sonographic confirmed arthritis were taken into account, prevalence of chronic arthritis would decline to 33.7%. Synovitis, which is one of the criteria to define pCHIKV-CIJD, was the most frequent sign, found in 65.5% of patients with abnormal US results. Similarly, in another study that evaluated hands and wrists of 50 patients with chronic musculoskeletal symptoms secondary to CHIKF in Brazil, synovitis was the most common finding (84.0%) [35]. Furthermore, a recent study confirmed that US could be of great value in confirming clinical findings in CHIKF patients, with the advantage of being a non-invasive, low-costing, and accessible exam [36].

Whatever may be the exact proportion of pCHIKV-CIJD, it brings major consequences to a patient’s QOL, as it significantly affects their mobility and mental health in a long-term way [32,37]. For example, a cross sectional study performed in northern Brazil has shown that CHIKF and RA groups, with comparable disease activity score (moderate), reported very similar perceptions of disease’s impact on their QOL, due to pain, mobility dysfunction, effect on daily activities and anxiety/depression [33]. Conversely, significantly more RA patients took steroids and methotrexate, which may indicate that CHIKF patients are undertreated due to lack of robust evidence of benefits.

As already found in other studies [29,32], in our cohort the univariate analysis shows that females had worse prognosis. Although some studies have found that age can be determining factor for chronification [12,38], in our population this finding could not be reproduced. However, the number of patients in the pCHIKV-CIJD group was limited for age stratification. It was not possible to observe the influence of pre-existing rheumatic diseases either, as previously described elsewhere [12], as only one patient in chronic group reported this condition.

None of the symptoms or signs presented at D0 was predictive of chronification. Conversely, patients who remained symptomatic beyond the acute phase (at D21) presented increased risk of pCHIKV-CIJD. These clinical findings seem to be supported by some studies that described immune response during CHIKF evolution. Acute phase is remarkably characterized by proinflammatory cytokines, including IFNα, which is a strong mediator of innate antiviral response, and also IFN γ, IL-2, IL-2R, IL-6, IL-7, IL-12, IL-15, IL-17 and IL-18, which could be demonstrated across different cohorts in a meta-analysis [39]. Notwithstanding, immune mediator profile change over time during CHIKF evolution, and the persistence of some cytokines, such as IL-6 and IL-12, correlates to chronic disease [40,41]. Changing inflammation patterns could explain different clinical manifestations along evolutive phases of the disease, and patients who develop chronic arthritis may probably exhibit contrasting patterns with those who recuperate earlier.

In a small cohort of 10 patients with confirmed CHIKF, followed for 12 months, when the six patients whose evolution was classified as sub-acute or chronic were compared to the four who resolved all symptoms before day 10, IL-6 behavior was significantly different (p<0,05). In those volunteers who acutely resolved symptoms, mean IL-6 was slightly above the upper limit of normality (ULN) only at the time of diagnosis, but was already normal in day 10, and also after three months. In contrast, in sub-acute and chronic cases, mean IL-6 was more than five times above the ULN at the time of diagnosis and persisted like that for 3 months or more [42].

Correspondingly, Hoarau and colleagues [43] evaluated a large set of immunological and biochemical lab parameteres from 32 hospitalized patients with confirmed CHIKF, in whom they observed a robust cellular and molecular innate immune response during acute phase in all patients, characterized by activaction of NK cells, plasmocytoid dendritic cells and T cell subsets, as well as higher serum levels of IFNγ and IL-12 when CHIKF patients were compared to those with other infections and to healthy controls. A subgroup analysis compared six patients who fully recovered from acute CHIKF to nine who experienced chronic arthralgia 12 months after infection. Interestingly, no difference regarding Th1 or Th2 cytokines levels was observed between the groups during acute phase. However, IL-12, an essential cytokine to initiate NK cells and macrophages activation in response to infectious challenges, as well as estimulate the development of a Th1 profile with a potent antiviral activity, had a sharply different behavior: while dramatically elevated in both groups in acute phase, in subacute phase (after 15 days) it returned to background levels exclusively in recovered patients, but persisted elevated in those with chronic joint pain. The authors advocate that this pattern could be indicative of continuous stimulation of the innate immune cellular response in patients prone to chronic disease, perhaps because of viral or antigenic persistence in immunoprivileged niches. Even though these mechanisms are not completely understood, immune response and inflammation apparently diverge between patients who recover and those who chronify at some point after acute phase.

Edema was a prominent sign in predicting chronification of disease, either when reported by the patient or when observed during physical examination, probably acting as a clinical marker of more intense inflammatory activity in articular microenvironment. One previous study has also pointed out to early edema as a risk factor for persistent arthralgia for longer than 12 months (OR = 13.46), along with hypertension, retro-ocular pain, sex and age. The authors built a clinical score that was able to distinguish individuals into high or low risk of chronic arthralgia, with an overall accuracy of 76%, sensitivity of 94% and specificity of 58% [34].

Although it has been very sensitive, the score lacks specificity if its goal would be the selective indication of early pharmacological interventions. Moreover, it is pertinent to discuss if either the acute or the post-acute phase of CHIKF would be the best moment to apply this score. Our cohort pointed out not only that remaining symptomatic in the post-acute phase positively correlates to chronification, but also spotted an expressive proportion of patients who reported and/or exhibited edema at D21 visit though had not manifested this sign at the inclusion visit. Aforementioned studies of immune response corroborate the hypothesis that the turning point of incessant inflammation occurs later in the course of the disease.

Different virus genotypes have also been suggested as an explanation for the disparity in the proportion of patients with poorer prognosis in different cohorts. A systematic review showed that the ECSA-diverged/ Indian Ocean Lineage (IOL) genotype seems to generate chronic cases more often (50%) [27]. In our study, as in other studies carried out in Brazil [33,44–47], the genotype was ECSA and, although the percentage of patients with pCHIKV-CIJD exceeded 45%, we did not find any unique mutations that could be related to prognosis. Similarly, we were not able to demonstrate correlation of early CHIKV viral load with chronic disease, despite previous studies which had shown it before [39,40].

Our study has the limitation that almost half of the volunteers have lost the follow-up, resulting in small numbers of subjects in each comparative groups. This might be the reason for not having confirmed some findings of previous cohorts. Furthermore, it is very reasonable to suppose that clinical manifestations alone have insufficient capacity to distinguish precisely patients that would potentially benefit from early treatment. Albeit the utility of inflammatory biomarkers is yet to be defined in CHIKV-derived chronic arthritis, their addition to clinical scores has the potential to improve accuracy. This risk-based approach would allow researchers to investigate whether the early implementation of specific therapeutic strategies, such as methotrexate or disease-modifying antirheumatic drugs, could prevent the incapacitating form of the disease, permanent joint damage and impact in QOL.

In conclusion, this cohort has provided important insights into the prognostic evaluation of CHIKF. Symptomatic sub-acute disease is a relevant predictor of evolution to chronic arthritis with synovitis, drawing attention to joint pain, edema, multiple articular involvement including small hand and feet joints as risk factors for chronification beyond three months, especially in women. Future studies are needed to accomplish the identification of accurate and early biomarkers of poor clinical prognosis, which would allow better understanding of the disease’s evolution and improve patients’ management, modifying CHIKF burden on global public health.

## Supporting information

S1 Fig Symptoms and signs evolution throughout the follow-up. **A.** Symptoms and signs reported at inclusion (D0), 21-day follow-up (D21), and 90-day follow-up (D90) visits. Each symptom is represented in a circle and a color. The size of the circles represents the number of volunteers who had that symptom. Each line connecting two symptoms represents that they occur simultaneously, and the line thickness represents the number of volunteers who had that combination of symptoms. **B.** Joints most often affected by pain. Black bars on the left represent the number of volunteers who reported pain in that joint. Black lines and dots represent pain in all the hachured joints simultaneously in a volunteer, and the upper bars represent the number of volunteers who had that intersection. Intersections of affected joints that occurred in less than two volunteers were removed from the image for simplification. Green area highlights upper joints and orange area represents lower joints.
(TIF)

S2 Fig Affected joints and sonographic findings in ultrasonography examination. **A.** Most often affected joints. **B.** Most common sonographic findings. The horizontal bars on the left represent the number of volunteers with abnormalities in each joint (A) or the type of sonographic alteration (B). Lines connecting black dots represent joint involvement (A) or type of alteration (B) observed simultaneously in a volunteer, and the upper vertical bars represent the number of volunteers who had that intersection. Green areas highlight upper joints and orange areas represent lower joints.
(TIF)

S1 Table Clinical, laboratory and follow-up information of the 169 CHIKF-confirmed volunteers. (XLSX)

S2 Table Continuous variables and univariate analysis from CHIKF-confirmed patients at D0 and D21. (XLSX)

S3 Table Clinical categorical variables and univariate analysis from CHIKF-confirmed patients at D0, D21 and D90. (XLSX)
