# Supplementary figures and images for: Clinical markers of post-Chikungunya chronic inflammatory joint disease: A Brazilian cohort
Source: PLoS Negl Trop Dis. 2023 Jan 6;17(1):e0011037. doi: 10.1371/journal.pntd.0011037 (PMC9851532; doi:10.1371/journal.pntd.0011037)

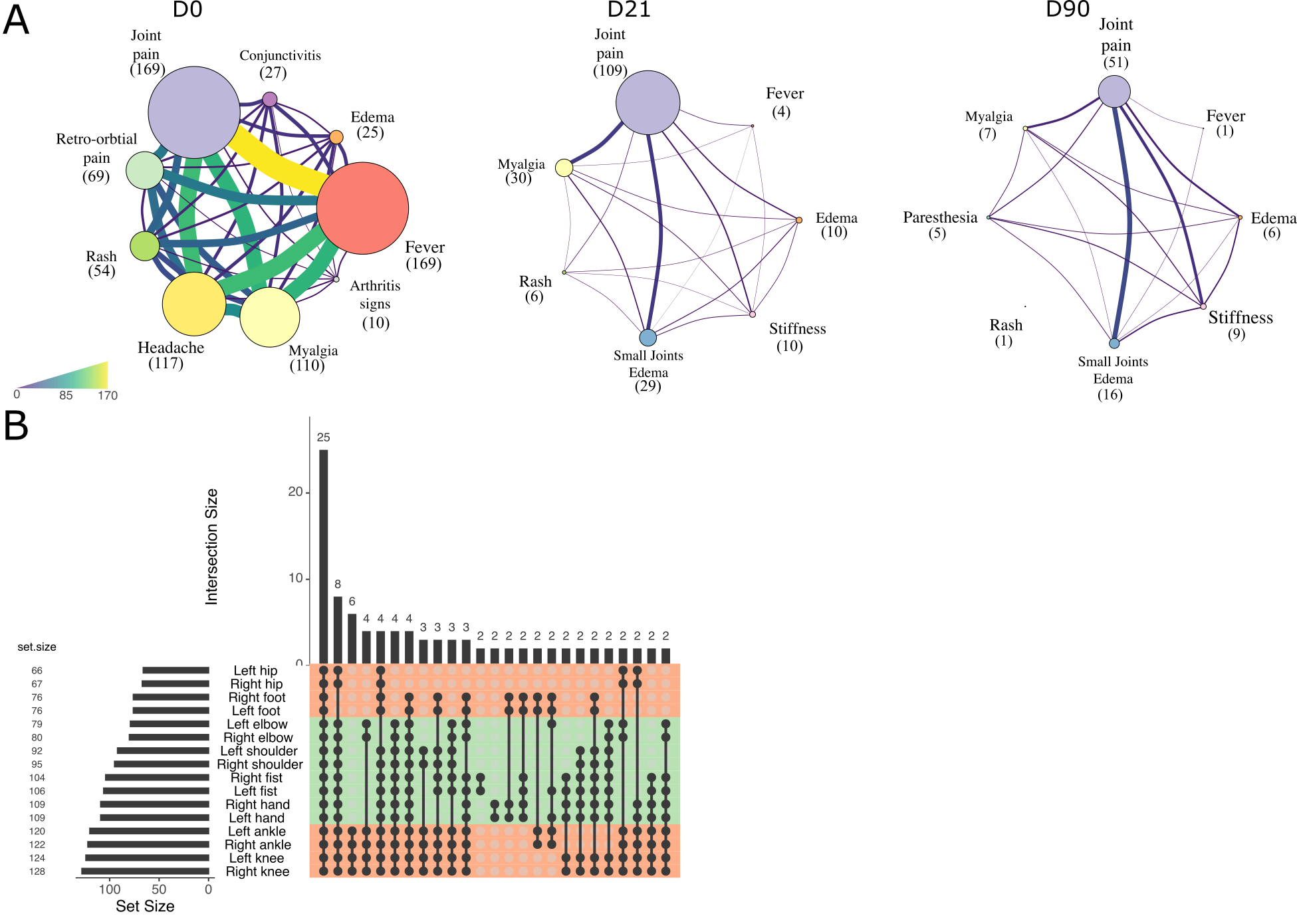

Supplement: S1 Fig — A. Symptoms and signs reported at inclusion (D0), 21-day follow-up (D21), and 90-day follow-up (D90) visits. Each symptom is represented in a circle and a color. The size of the circles represents the number of volunteers who had that symptom. Each line connecting two symptoms represents that they occur simultaneously, and the line thickness represents the number of volunteers who had that combination of symptoms. B. Joints most often affected by pain. Black bars on the left represent the number of volunteers who reported pain in that joint. Black lines and dots represent pain in all the hachured joints simultaneously in a volunteer, and the upper bars represent the number of volunteers who had that intersection. Intersections of affected joints that occurred in less than two volunteers were removed from the image for simplification. Green area highlights upper joints and orange area represents lower joints. (TIF) [file pntd.0011037.s001.tif]

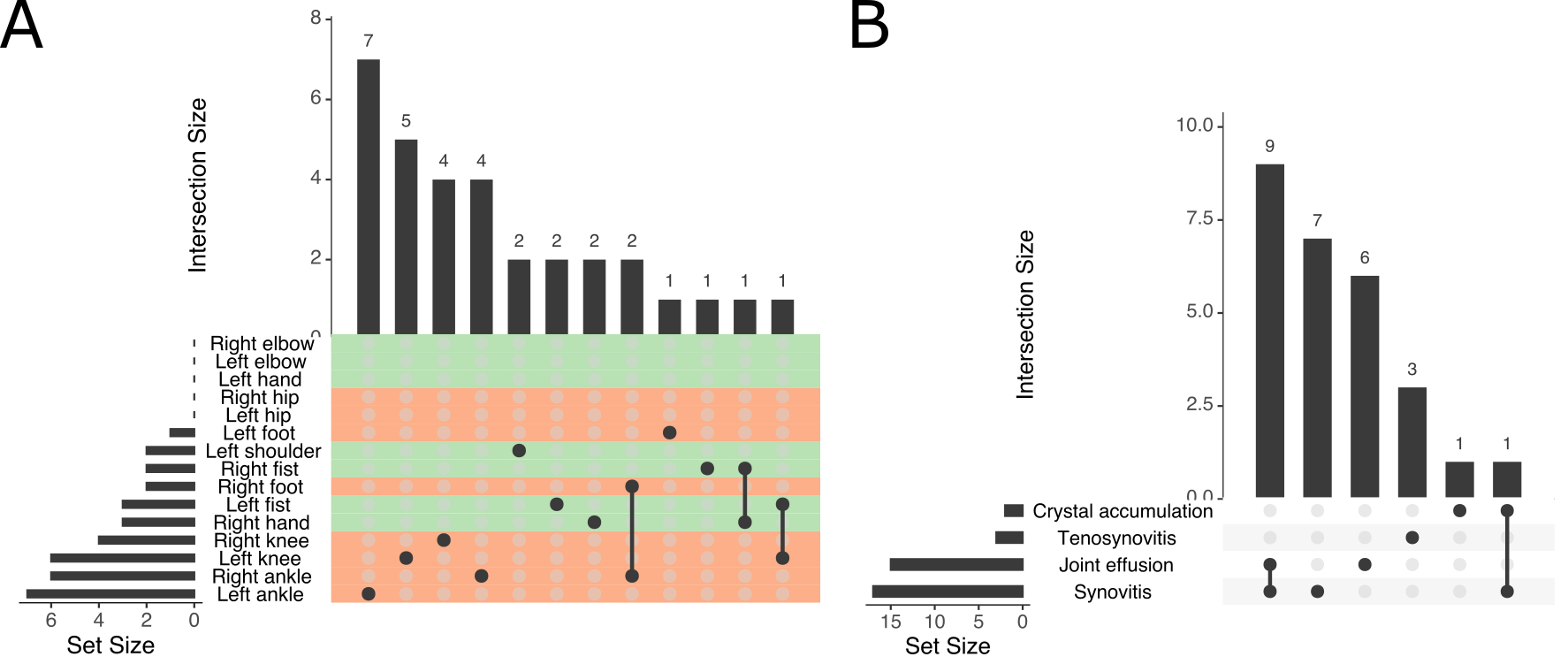

Supplement: S2 Fig — A. Most often affected joints. B. Most common sonographic findings. The horizontal bars on the left represent the number of volunteers with abnormalities in each joint (A) or the type of sonographic alteration (B). Lines connecting black dots represent joint involvement (A) or type of alteration (B) observed simultaneously in a volunteer, and the upper vertical bars represent the number of volunteers who had that intersection. Green areas highlight upper joints and orange areas represent lower joints. (TIF) [file pntd.0011037.s002.tif]
